# Rare-Earth Elements Extraction from Low-Alkali Desilicated Coal Fly Ash by (NH_4_)_2_SO_4_ + H_2_SO_4_

**DOI:** 10.3390/ma16010006

**Published:** 2022-12-20

**Authors:** Andrei Shoppert, Dmitry Valeev, Julia Napol’skikh, Irina Loginova, Jinhe Pan, Hangchao Chen, Lei Zhang

**Affiliations:** 1 Department of Non-Ferrous Metals Metallurgy, Ural Federal University, Yekaterinburg 620002, Russia; 2 Laboratory of Sorption Methods, Vernadsky Institute of Geochemistry and Analytical Chemistry, The Russian Academy of Sciences, Moscow 119991, Russia; 3 Key Laboratory of Coal Processing & Efficient Utilization, Ministry of Education, School of Chemical Engineering and Technology, China University of Mining & Technology, Xuzhou 221116, China

**Keywords:** coal fly ash, rare-earth elements, acid leaching, leaching kinetics, extractive metallurgy, waste utilization

## Abstract

Coal fly ash (CFA) obtained from pulverized coal furnaces is a highly refractory waste that can be used for alumina and rare-earth elements (REEs) extraction. The REEs in this type of CFA are associated with a mullite and amorphous glassy mass that forms a core-shell structure. In this research, it was shown that complete dissolution of amorphous aluminosilicates from the mullite surface with the formation of the low-alkali mullite concentrate prior to sulfuric acid leaching with the addition of (NH_4_)_2_SO_4_ helps to accelerate the extraction of REEs. The extraction degree of Sc and other REEs reaches 70–80% after 5 h of leaching at 110 °C and acid concentration of 5 M versus less than 20% for the raw CFA at the same conditions. To study the leaching kinetics of the process, the effects of temperature (90–110 °C), liquid-to-solid ratio (5–10), and leaching time (15–120 min) on the degrees of Al and rare-earth elements (REEs) extraction were evaluated. After 120 min of leaching at 110 °C and L/S ratio = 10, the extraction of Al was found to be lower than 30%. At the same time, total REEs (TREE) and Fe extraction were greater than 60%, which indicates that a part of the TREE was transferred into the acid soluble phase. After leaching, the residues were studied by laser diffraction (LD), X-ray diffraction (XRD), X-ray fluorescence (XRF), and scanning electron microscopy (SEM-EDS) to evaluate the leaching mechanism and the solubility of Al- and Fe-containing minerals, such as mullite, hematite, and amorphous aluminosilicate.

## 1. Introduction

Coal fly ash (CFA) or coal combustion residuals (CCRs) are the waste left after coal combustion in thermal power plants (TPP). CFA is one of the largest industrial waste streams in the world. Russia accumulates ~20 Mt per year of CFA, and its global generation is estimated to be several billion tons annually [1]. Major large-scale applications of fly ash include using it as a building material for construction (e.g., concrete and geopolymer cement, road base, ceramics, etc.) [2,3,4,5], but the degree of ash that is used in the industry is still low, especially in the developing counties. For example, one of the largest power plants of Russia, the Reftinskaya TPP produces about 4 Mt of CFA annually [6], of which only 6% is consumed by the construction industry (about 0.3 Mt per year), while the rest is stored in CFA landfills.

CFA can lead to both air pollution (in dust) and soil contamination and it is hazardous to human health [7,8]. The waste product causes environmental pollution primarily due to the adverse chemical characteristics of the ash and the content of heavy metal elements such as Cd, Cr, Pb, As, and Hg [5]. However, the main components of fly ash (oxides of silicon, calcium, aluminum, and iron) and higher levels of rare-earth elements (REEs) make its recycling economically and environmentally beneficial.

The REEs content in Russian CFA is around 400 ppm, which is generally about that of raw bauxite for alumina production but lower than in the bauxite residue red mud (RM), which is often considered as a technogenic source of REEs [9,10,11]. The RM utilization is complicated by a high content of alkali, requiring additional costs for its neutralization [12]. However, the CFA recycling does not require special conditions. The main minerals can be dissolved at atmospheric pressure by hydro-metallurgical methods due to the high solubility of silicates in NaOH. This fact facilitates the extraction of REEs and other elements (such as Al or Fe) [13,14,15].

The main method of CFA treatment in the world is their leaching with acidic reagents, which can be used both as mineral acids and as organic cation exchangers [15]. According to research reports, the REEs in the CFA are usually encapsulated in the aluminosilicate glassy phase, and therefore their dissolution during acid leaching is difficult [16,17,18,19,20]. To extract the REEs, it is necessary to study the influence of preliminary activation of the CFA that would allow selective extraction of REEs without transferring Si, Al, and Fe into a solution.

The extraction of REEs by direct nitric acid leaching at T = 85–90 °C did not exceed 44% [21], while pretreatment by lime roasting can increase the REEs extraction to 80% or more [22]. The high efficiency of REEs extraction is associated with an increase in the solubility of Al minerals, which can be associated with REEs in CFA. A strong positive correlation between the REEs and the Al plus Si contents was shown in analysis of the chemical composition of the different size fractions of a class-F fly ash [23,24].

Previous studies have shown that alkaline leaching is a widely used hydrometallurgical activation method for enhancing acid leaching recovery of REEs [25,26]. Dependence of extraction of light or of heavy REEs on the parameters of alkali treatment was also observed in studies of Western Kentucky No. 13 and fire clay coal wastes [27]. However, due to the dissolution of Si and the presence of sodium aluminate in the solution, the process of the desilication product (DSP) precipitation occurs. This leads to the high concentration of Na_2_O (up to 14%), Al and other impurities that precipitate together with the desilication product [28,29]. High concentration of alkali greatly increases acid consumption in the further processes of Al and REEs extraction. Therefore, stepwise desilication is significant in the synergistic utilization of CFA with the simultaneous extraction of Si, Al, and REEs [26,30,31].

In our previous research, it was found that under certain leaching conditions, it becomes possible to retain silica in solution due to its presence in the metastable zone. This makes it possible to obtain raw materials for the alumina industry with the low alkaline content [28]. According to preliminary analyses, REEs are concentrated in the solid residue after leaching [32].

In this article, the REEs and basic metals extraction from pulverized coal (PC) furnace fly ash after a novel method of preliminary desilication to optimize the leaching process was studied. Since sulfuric acid is less volatile and more cost-effective compared with the other acids, 50% sulfuric acid with the addition of (NH_4_)_2_SO_4_ was chosen as the leaching agent. The effects of temperature, liquid-to-solid ratio, and leaching time on Al, Fe, and REEs extraction were examined. The leaching mechanisms and kinetics were studied using the shrinking core model, XRD, and SEM-EDS methods.

## 2. Materials and Methods

### 2.1. Materials and Reagents

**Figure 1:**
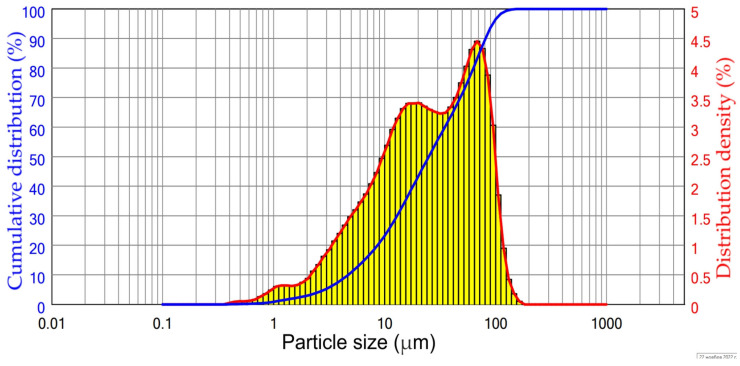
The particle size distribution of the raw CFA.

The CFA used in this study was collected from the Reftinskaya TPP in Asbest, Russia. This plant uses pulverized furnace for coal combustion at temperatures higher than 1100 °C. The particle size distribution of the raw CFA is shown in Figure 1. The chemical composition of the CFA is shown in Table 1. According to Table 1 and Figure 1, the raw CFA has a typical CFA class F composition with a high amount of SiO_2_ (>60%) and a low amount of Al_2_O_3_ (<30%). The median particle size of the CFA was about 25 μm. Before leaching experiments, the CFA was subjected to magnetic separation based on a procedure described elsewhere [33]. The NaOH leaching process used to obtain DCFA was carried out according to our previous research [29] at T = 110 °C, L/S ratio = 10, 11.6 mol L^−1^ of NaOH, and a leaching time of 25 min.

### 2.2. Analysis

The mineral and chemical compositions of the raw CFA and the solid residues after NaOH and acid leaching were evaluated using X-ray diffraction (XRD) on a Difrei-401 diffractometer (JSC Scientific Instruments, Saint Petersburg, Russia) and X-ray fluorescence (XRF) method on an Axios MAX X-ray fluorescence spectrometer (Malvern Panalytical Ltd., Almelo, The Netherlands). The diffractometer was equipped with a Cr-Kα radiation source with a 2θ range from 15° to 140° and an exposure time of 30 min. The operating mode of the X-ray source was set to 25 kW/4 mA. The mineral phases in the raw material and the solid residues were analyzed using «Match 3» software. The concentration of REEs in the solid was measured after complete dissolution of the solid residue by a mixture of concentrated hydrofluoric, sulfuric, and nitric acids. After leaching by acid mixture, the residue was fused with soda and boric acid at 950 °C and then leached with 1 N HCl. The resulting solutions and leachates from the experiments that were diluted with 5% HNO_3_ were used to determine the REEs concentration by inductively coupled plasma optical emission spectrometry (ICP-OES) analysis on a spectrometer Vista Pro (Varian Optical Spectroscopy Instr., Mulgrave, Australia). The procedure of the sequential chemical extraction that was used to determine the REEs phase distribution is described elsewhere [23].

The surface morphology and elemental composition of the CFA and the solid residues after NaOH and acid leaching were investigated using scanning electron microscopy with energy-dispersive X-ray spectroscopy (SEM-EDX, Vega III, Tescan, Brno, Czech Republic).

The particle size distribution and specific surface area of the samples were determined via laser diffraction method (LD) using Bettersizer ST (Bettersize Instruments Ltd., Dandong, China) and Brunauer–Emmett–Teller method (BET) using NOVA 1200e (Quantachrome Instruments, Boynton Beach, FL, USA), respectively. Before BET analysis, all samples were subjected to degassing under vacuum at 200 °C for 12 h.

### 2.3. Experiment

Leaching by NaOH or a mixture of (NH_4_)_2_SO_4_ and H_2_SO_4_ was conducted in a thermostated 1 L stainless steel reactor and a thermostated 0.5 L glass reactor, respectively. The reactors have chemical reagent additions opening, temperature control mechanisms, and a water-cooled condenser for the recycling of evaporated water. The stirring speed in all experiments was 400 rpm, which was sufficient to keep the particles in suspension. Briefly, 50 g of CFA was added to a 500 mL of the solution with a NaOH concentration of 11.6 mol L^−1^ and temperature of 110 °C. The 30, 45 and 60 g of the desilicated coal fly ash (DCFA) was added to a 300 mL solution containing 1 mol L^−1^ (NH_4_)_2_SO_4_ and 7 mol L^−1^ H_2_SO_4_. The results of Xu et al. [34] and Valeev et al. [35] show that the concentration of H^+^ higher than 14–15 mol L^−1^ do not lead to further increases in Al extraction from CFA and high-silica bauxites. After the desired leaching time (15–300 min) the pulp was filtered, and the solid residue was dried at 110 °C for 240 min before analysis using ICP-OES.

The extraction degree of REEs from CFA after NaOH and DCFA after acid leaching was calculated by the Equation (1):

α = [(m_1_ × Me_1_)/(m_2_ × Me_2_)] × 100%, (1)

where Me_1_ is the REE content in the solid residue obtained after the raw material leaching by NaOH or acid, %; m_1_ is the weight of the solid residue, Me_2_ is the content of REE in the raw material, %; m_2_ is the weight of the raw material load in the experiment, g.

## 3. Results and Discussion

### 3.1. Raw Materials Characterization

**Figure 2:**
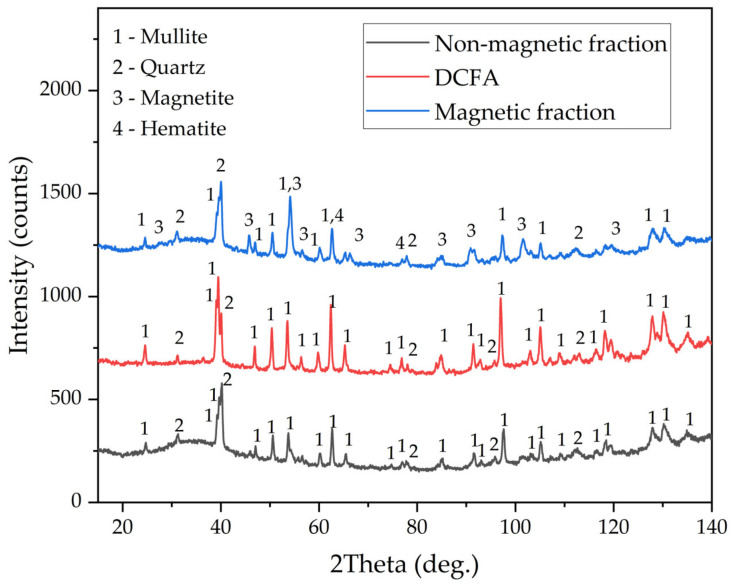
XRD patterns of the raw CFA, magnetic fraction obtained by magnetic separation, and DCFA.

The chemical compositions of the magnetic fraction, non-magnetic fraction before desilication, and DCFA are shown in Table 2. The X-ray diffraction pattern of the non-magnetic fraction and DCFA, as well as magnetic fraction samples, are shown in Figure 2. The mineral composition of CFA (non-magnetic fraction) includes quartz (SiO_2_), mullite (3Al_2_O_3_·SiO_2_), and an amorphous glassy mass (from 20° to 50° 2θ). After desilication, an amorphous glassy mass was completely dissolved, as confirmed by the XRD pattern of DCFA. The main crystalline phase of the magnetic concentrate is magnetite (Fe_3_O_4_). However, there is also a high amount of mullite and quartz. These phases may be included in the solid matrix of the magnetite or physically entrapped within the concentrate. The chemical composition of the magnetic fraction confirms this, with a high amount of SiO_2_ and Al_2_O_3_ seen. Nevertheless, the content of Fe_2_O_3_ in the concentrate is four times higher than in the raw CFA. The yield of the magnetic fraction is 8.5 wt.%. The chemical compositions of the non-magnetic fraction are almost identical to those of the CFA except for iron content, which was reduced from 3.43 wt.% to 1.58 wt.%.

**Figure 3:**
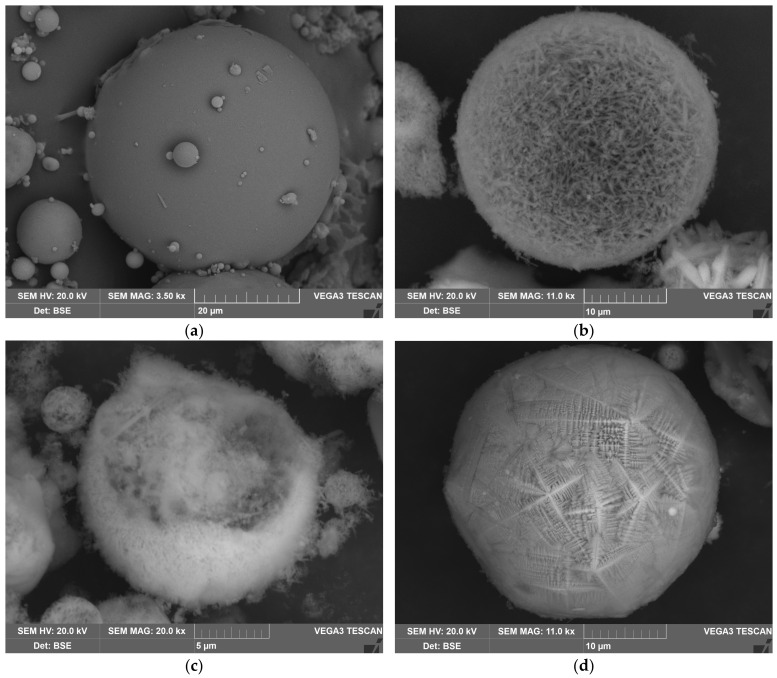
The SEM images of the CFA aluminosilicate particles surface at 3500 magnitude (**a**); the SEM images of the agglomerates of the mullite particles at 11,000 magnitude (**b**); the SEM images of the agglomerates of the mullite particles at 20,000 magnitude (**c**); and magnetitic particles at 11,000 magnitude (**d**).

As shown in Table 2, the Al_2_O_3_ content in DCFA was increased to 42.01 wt.%, while Na_2_O concentration was maintained at the same level (0.85 wt.%). This means that during the novel fast method of desilication, there is no DSP formation, and all the amorphous glassy mass is completely removed. The results of the BET analysis (Table 3) of the non-magnetic fraction and DCFA and SEM images in Figure 3 support the observation.

The results of the BET analysis of the non-magnetic fraction and DCFA are shown in Table 3. After desilication, the specific surface area of DCFA increased more than 3 times and the total pore volume increased 3.5 times.

To demonstrate the reason for these differences, the SEM images of the raw CFA and DCFA are provided (Figure 3). The CFA is presented by smooth spheres of mullite covered by glassy mass. After desilication, spherical agglomerates of acicular mullite particles with high porosity can be seen in DCFA. In the magnetic concentrate, there are magnetite spheres composed of crystals with an orientation along an axis.

The output of DCFA at the NaOH leaching parameters presented in Section 2.3 was 52.5 wt.%. It means that Al_2_O_3_ content should be doubled; however, amorphous glassy mass also has Al in its chemical composition [36]. Therefore, 17.6% of Al_2_O_3_ was dissolved during NaOH leaching. The extraction degree of SiO_2_ was 66.4%. The minor components concentration determined by ICP-OES analysis of the solids is also shown in Table 2. The REEs in this type of CFA are mainly presented by Sc, Y, and light REEs such as La, Ce, and Nd and these elements are concentrated in the non-magnetic fraction, which is consistent with previous research [18,37].

The concentration of REEs in DCFA was found to be almost doubled, which suggests that they are not dissolved in NaOH. It can be explained that in the alkaline conditions, according to [37], REEs that were dissolved together with the amorphous glassy mass are precipitated in the hydroxide form (Equation (2)).

REE^3+^ + 3OH^−^→REE(OH)_3_↓, (2)

**Figure 4:**
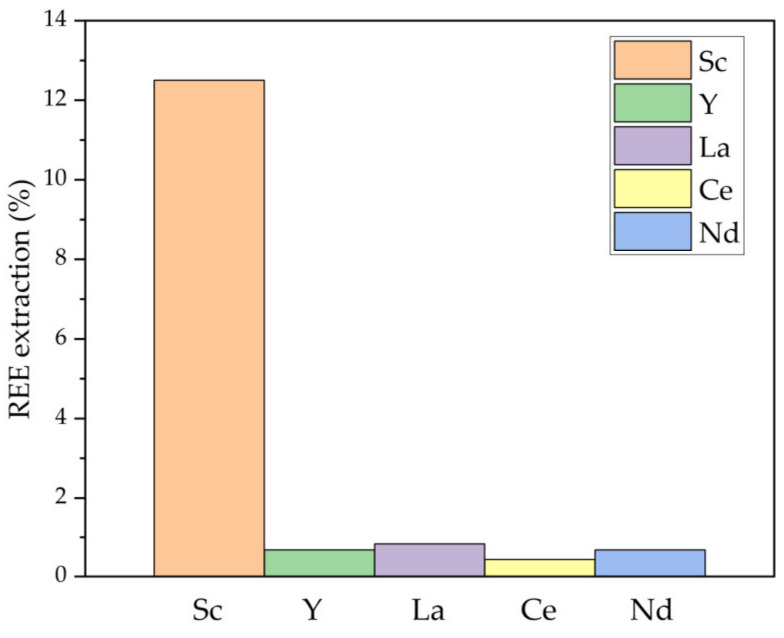
The extraction degree of REEs from non-magnetic fraction by NaOH leaching at 110 °C, L/S ratio = 10, C_NaOH_ 11.6 mol L^−1^, and leaching time 25 min.

The REEs extraction by NaOH is shown in Figure 4. Alkaline solutions can still dissolve Sc, which was also found by Li et al. [27].

**Figure 5:**
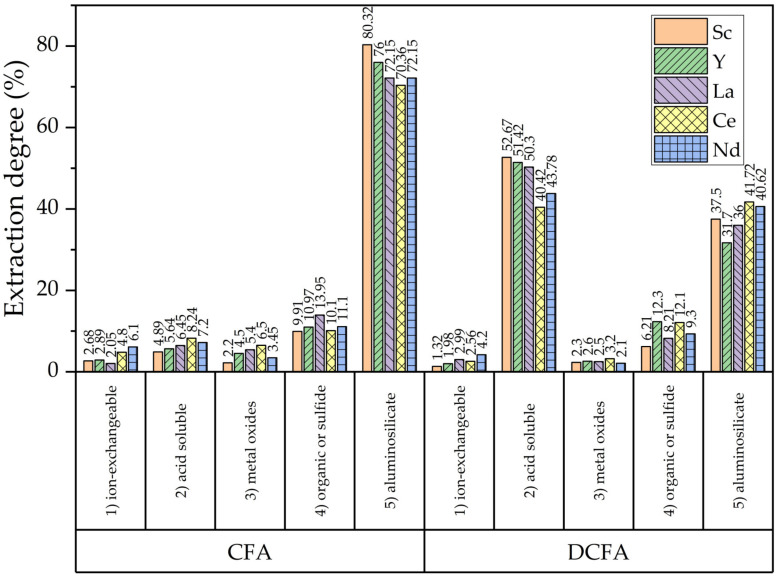
The results of the sequential chemical extraction procedure to study the mode of occurrences of REEs in CFA and DCFA.

After the precipitation of REEs from alkaline solutions, the proportion of acid-soluble phases should be expected to increase. To investigate the distribution of REEs in CFA before and after NaOH leaching, a sequential chemical extraction procedure [15] (Figure 5) was conducted. The NaOH leaching helps to increase the amount of acid-soluble phase by dissolving the aluminosilicates phases. However, the achieved results are lower than those obtained using PCB fly ash and standard desilication after 2 h or more [37,38,39]. Another contributing factor to the higher extraction from DCFA is the higher specific surface area of the particles (Table 3).

**Figure 6:**
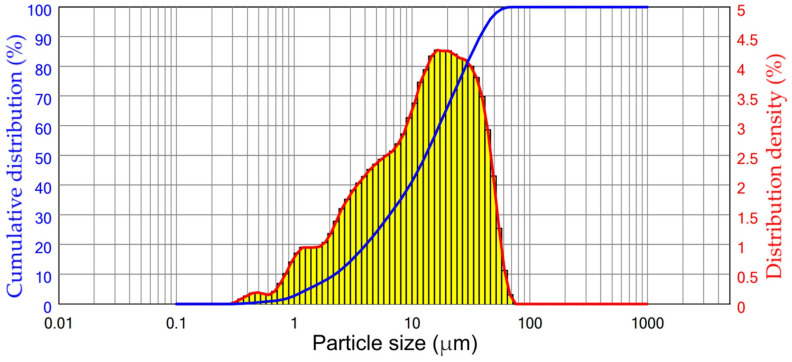
The particle size distribution of DCFA used in the REEs leaching experiments.

The particle size distribution of DCFA used to study the effect of different leaching parameters on the extraction of valuable components and kinetics of leaching is shown in Figure 6. The particle size distribution of the DCFA indicates that NaOH leaching leads to a significant reduction in particle size in comparison with the raw CFA, which can be explained by amorphous glassy mass dissolution from the surface of the particles. The median particle size of the DCFA was 13.1 μm.

### 3.2. (NH_4_)_2_SO_4_ + H_2_SO_4_ Leaching

**Figure 7:**
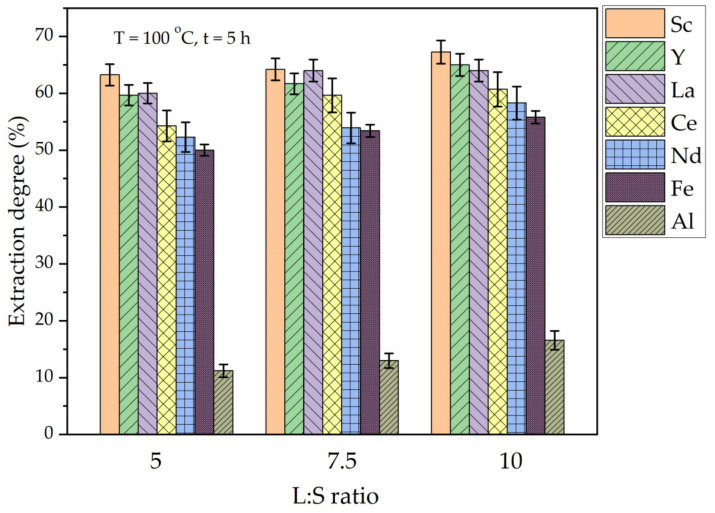
The effect of L/S ratio on the extraction degree of REEs, Fe, and Al from DCFA by (NH_4_)_2_SO_4_ + H_2_SO_4_.

**Figure 8:**
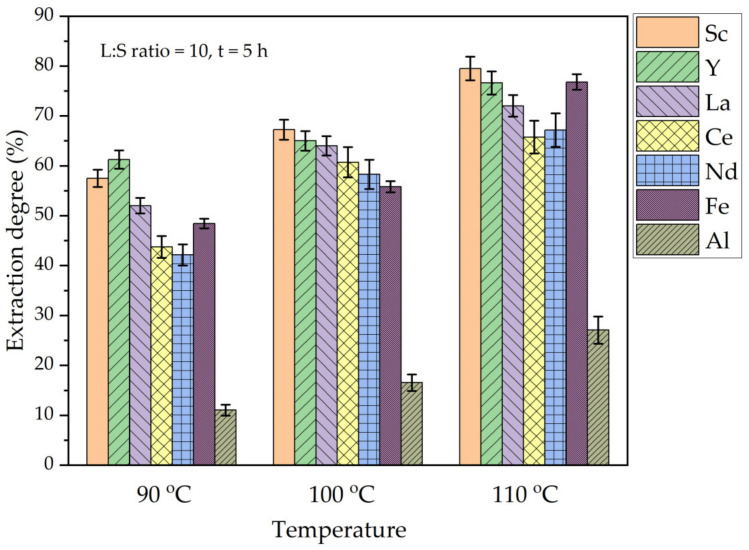
The effect of temperature on the extraction degree of REEs, Fe, and Al from DCFA by (NH_4_)_2_SO_4_ + H_2_SO_4_.

To reduce the amount of acid required for Al extraction from CFA and high-silica bauxite, while maintaining a high Al and REEs extraction, an ammonia/sulfuric acid leaching method was suggested [35,40]. In addition, the presence of ammonia sulfate helps to eliminate organic carbon preg-robbing effect, because it is used for the REEs desorption process [41]. In this study, the effect of leaching time and L/S ratio on the amount of Al and REEs that were extracted from DCFA by a mixture of 1 mol L^−1^ (NH_4_)_2_SO_4_ and 7 mol L^−1^ H_2_SO_4_ was studied (Figure 7 and Figure 8). This concentration of components in solution was chosen because the results of Xu et al. [34] showed that concentrations of H^+^ higher than 14–15 mol L^−1^ do not lead to further increase in Al extraction. However, the effect of acid excess was evaluated in this research by varying the L/S ratio from 5 to 10 at a temperature of 100 °C and a leaching time of 5 h (Figure 7).

Figure 7 shows that an REEs extraction more than 50% was achieved at all the L/S ratios after 5 h of leaching at 100 °C. However, the Al extraction was lower than 20% at these conditions. The increase in the L/S ratio from 5 to 10 leads to an increase in the extraction of all the elements by 5–7%. Therefore, the L/S ratio has a little effect on the REEs extraction from DCFA under atmospheric pressure. To enhance the extraction of Al, the high-pressure leaching method commonly is used [42], which implies a high effect of temperature on the mullite leaching.

As shown in Figure 8, the extraction of Al, Fe, and REEs significantly increased when the leaching temperature was increased from 90 °C to 110 °C. When the temperature is increased from 90 to 110 °C, the Sc extraction increases from 57 wt.% to almost 80 wt.%. The extraction of Fe and other REEs also greatly improved (to 65–75%) with the temperature increase. However, the Al extraction efficiency still does not exceed 30 wt.% at 110 °C, although there is a progress with the increase in temperature. Moreover, the increase in the Al extraction was in a linear relationship with the increase in REEs extraction. As a result, the REEs are extracted from the solid matrix of mullite without complete dissolution of Al. The kinetics study using the shrinking core model (SCM) was conducted to determine the mechanism of REEs leaching.

SCM models are widely used to describe the heterogenous process of porous materials leaching by liquid media [43]. There are three main SCM equations that are used to model the leaching of a raw material core that shrinks to the center of the particle, leaving an insoluble porous solid product after the chemical reaction (Equations (3)–(5)):

1 − 2/3X − (1 − X)^2/3^ = k_2_t, (3)

1 − (1 − X)^1/3^ = k_1_t, (4)

X = k_3_t, (5)

where X is the extraction rate (proportion), k_i_ is the apparent rate constant of the Equations (min^−1^), and t is the leaching duration (min).

**Figure 9:**
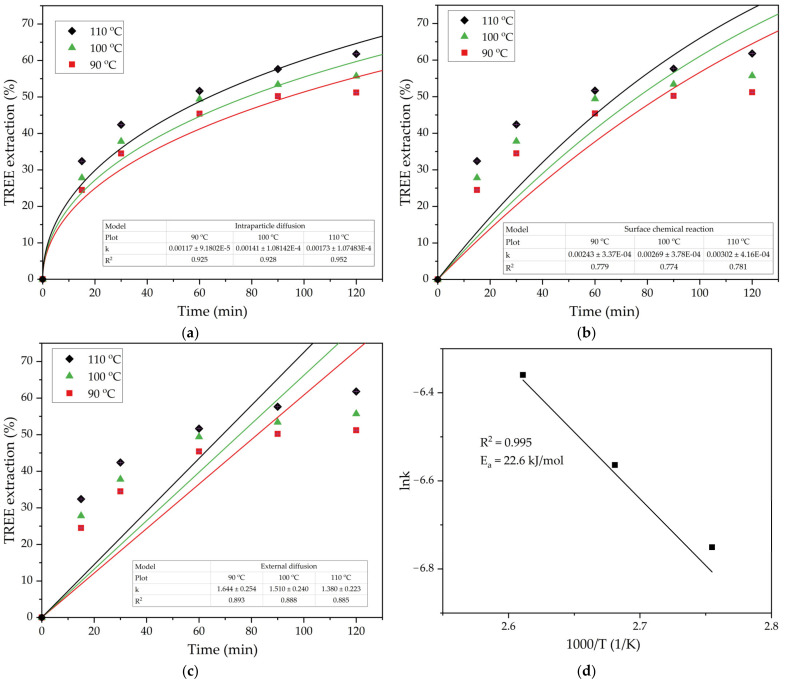
Results of fitting the experimental data (T = 90–110 °C, L/S ratio = 10) using the SCM model for the process limited by the diffusion through the solid product (**a**) by the surface chemical reaction (**b**) and by the diffusion through the liquid film (**c**) and the plot showing the dependence of lnk for the diffusion through the solid product on the inverse temperature (Arrhenius plot) (**d**).

Equation (3) is used to describe the process that is limited by the intraparticle diffusion through the solid product of the chemical reaction. Equation (4) is used to describe the process being limited by the surface chemical reaction, and Equation (5) is used to model the process limited by the diffusion through the liquid film (external diffusion). If the experimental data are better fitted to one of these equations, it means that this stage is a preferable limiting step of the process. The results of the effect of leaching time and temperature on the total REEs extraction (the sum of Sc, Y, La, Ce, and Nd extracted, TREE extraction) and the fitting of the data to the shrinking core model are shown in Figure 9.

Figure 9 indicates that the intraparticle diffusion Equation (3) was more suitable to describe the leaching process of TREE from the DCFA at all the temperatures. The solid products of reaction that controlled the rate of leaching were the silica-containing solid residue as well as the mullite that did not dissolve during the leaching process.

The experimental rate constants mentioned in Figure 9a were used to determine the energy of activation of the process by constructing a lnk − 1000/T plot (Figure 9d). Using the Arrhenius Equation (6) and the data in Figure 9d, the apparent activation energy (E_a_) of the leaching process can be determined as the slope of the straight line multiplied by the universal gas constant R.

k = k_0_ exp (−E_a_/RT), (6)

where k_0_ is the pre-exponential factor, E_a_ is the apparent activation energy (kJ mol^−1^), T is the reaction temperature (K), and R is the universal gas constant (8.314 J mol^−1^ K^−1^).

**Figure 10:**
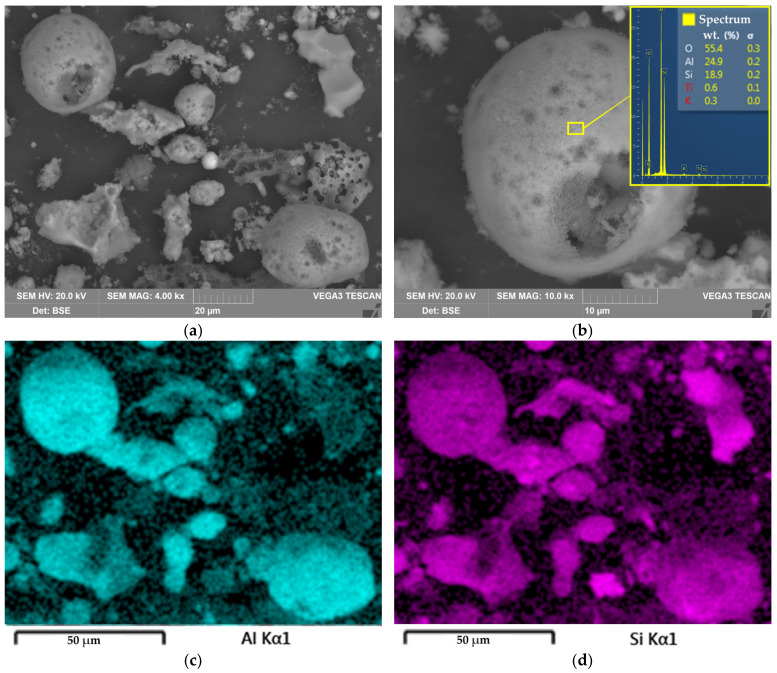
The SEM image of the solid residue particles surface at 4000 magnitude (**a**); the SEM image with EDX spectra of the mullite agglomerate at 10,000 magnitude (**b**); elemental distribution of Al on the surface of particles on Figure 10a (**c**); elemental distribution of Si on the surface of particles on Figure 10a (**d**).

The obtained value of the E_a_ 22.6 kJ mol^−1^ confirms that the diffusion is the limiting stage of the REEs extraction from DCFA. This was confirmed by the SEM-EDX analysis of the solid residue (Figure 10).

Figure 10 shows the SEM images of the solid residue obtained after the DCFA leaching at 110 °C, L/S ratio = 10, and leaching duration 5 h. As can be seen, the surface of the solid residue is covered by the caverns that remained after acid dissolution of the Al. Some particles were greatly corroded, and others were still in the form of spheres of mullite particles (Figure 10b). Therefore, the H^+^ ions and ions of the leached elements should diffuse through this aluminosilicate porous product to reach the core of the particles.

**Figure 11:**
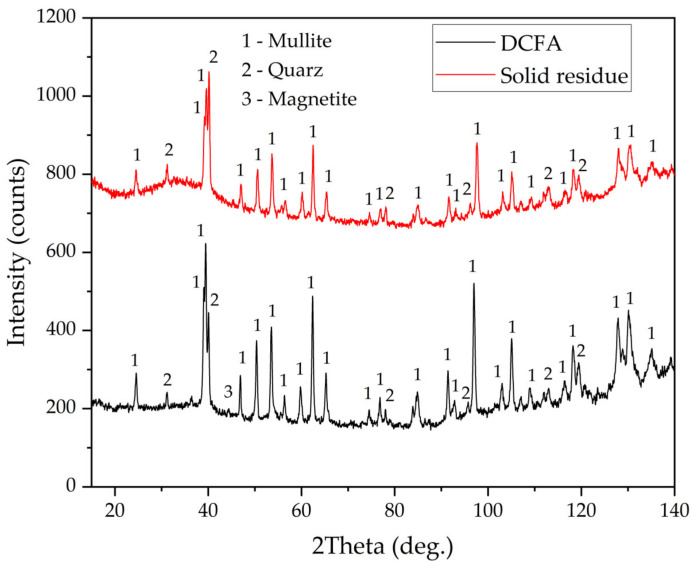
XRD patterns of DCFA and the solid residue obtained by the DCFA leaching at 110 °C, L/S ratio = 10, and leaching duration 5 h.

Furthermore, Figure 10c,d indicate that there are Al-depleted particles. These particles can be presented by quartz or precipitated amorphous silica, which formed during silicic acid hydrolysis [36]. The presence of amorphous silica can be confirmed by the XRD pattern of the solid residue, which is shown in Figure 11. After leaching, an amorphous phase can be seen from 20° to 50° 2θ. Additionally, the peaks of mullite after leaching were lowered, while the peaks of quartz became higher.

**Figure 12:**
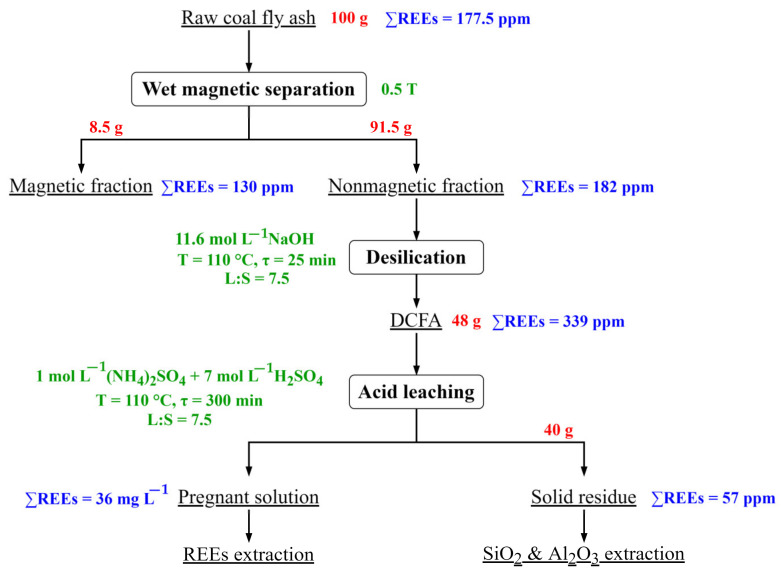
Flowsheet for the REEs extraction from the CFA.

Taking into account the obtained results, the following flowsheet of the REEs extraction from the CFA after desilication is proposed (Figure 12).

The adsorption and extraction are the most common method of the REEs recovery from the solution [44,45,46]. Therefore, our future research will be focused on the REEs recovery from the solution obtained via the leaching of DCFA at optimal conditions with the formation of the REEs concentrate using the adsorption method.

## 4. Conclusions

Coal fly ash is a promising source for REE extraction due to its abundance and high extraction values for REEs. The discrepancy in the characteristics of coal fly ash resulting from the coal combustion method leads to a high refractoriness of some materials. For example, the major minerals presented in the fly ash obtained from the pulverized furnace were mullite and quartz, which are insoluble by most acids and NaOH under atmospheric pressure. This resulted in a low extraction degree of REEs from such raw material. The desilication of CFA prior to acid leaching leads to the liberation of REEs from the solid matrix of the aluminosilicates, which increases the extraction degree. However, the convenient desilication method resulted in a high Na_2_O content in the desilicated product, which dramatically increased acid consumption.

In this article, a novel method of CFA desilication with a low content of Na_2_O in the product was used prior to REEs extraction by (NH_4_)_2_SO_4_ and H_2_SO_4_ mixture. The sequential leaching procedure showed that the amount of acid-soluble phase of REEs was increased after desilication to 40–52 wt.% vs 4–7 wt.% for the raw CFA. This can be explained by the dissolution of the amorphous glassy mass containing some part of the valuable elements, followed by the precipitation of REEs hydroxides from a NaOH solution. Furthermore, it was found that the solid concentration and temperature had a significant effect on REEs extraction. After 5 h of leaching at T = 110 °C and L/S ratio = 10, more than 80% of the selected REEs can be extracted. SEM-EDS images showed that preliminary desilication helps to completely dissolve the amorphous glassy mass from the surface of the spherical agglomerates consistent of acicular mullite particles. A high amount of porosity is formed when mullite is subjected to acid leaching, but the spheres still remain intact. Therefore, the aluminosilicate solid product of the leaching and the mullite can be a product layer that limits diffusion process.

The kinetic data obtained at different temperatures indicated that the leaching process follows the diffusion-controlled shrinking core model. The activation energy was determined using the obtained rate constants at different temperatures, and was found to be 22.6 kJ/mol. This confirms that the rate-limiting step of the process is diffusion. To further increase the extraction degree of REEs, high-pressure leaching or mullite particle dissolution can be used.

## Figures and Tables

**Table 1:** Chemical composition of the raw CFA.

Main Components, wt.%									
SiO_2_	Al_2_O_3_	CaO	Fe_2_O_3_	TiO_2_	MgO	Na_2_O	K_2_O	LOI	C
62.10	26.45	1.49	3.43	1.17	0.78	0.54	0.52	3.52	1.52

**Table 2:** The chemical composition of the magnetic fraction, non-magnetic fraction before desilication, and the DCFA.

Fraction	Main Components, wt.%									
	SiO_2_	Al_2_O_3_	CaO	Fe_2_O_3_	TiO_2_	MgO	Na_2_O	K_2_O	LOI	C
Magnetic fraction	50.86	24.19	2.53	13.87	0.92	0.89	0.22	0.67	2.78	0.78
Non-magnetic fraction	62.50	26.76	1.50	1.58	1.20	0.77	0.53	0.55	3.51	1.51
DCFA	40.00	42.01	2.94	3.41	2.32	0.87	0.85	0.08	7.20	3.10
**Fraction**	**Minor Components, mg kg^−1^**									
	Sc		Y		La		Ce		Nd	
Magnetic fraction	20		30		18		41		22	
Non-magnetic fraction	24		37		27		58		36	
DCFA	40		70		49		110		70	

**Table 3:** The BET analysis of the non-magnetic fraction and the DCFA after NaOH leaching at T = 110 °C, L/S ratio = 10, τ = 25 min.

Product	Specific Surface Area (BET) (m^2^/g)	Total Pore Volume (cm^3^/g)	Pore Diameter (nm)
Non-magnetic fraction	4.7	**8**	35.5
DCFA	15.1	28	18.7

## Data Availability

Not applicable.
